# Developmental changes in the association between approximate number representations and addition skills in elementary school children

**DOI:** 10.3389/fpsyg.2013.00783

**Published:** 2013-10-24

**Authors:** Jan Lonnemann, Janosch Linkersdörfer, Marcus Hasselhorn, Sven Lindberg

**Affiliations:** ^1^Department of Education and Human Development, German Institute for International Educational Research (DIPF)Frankfurt am Main, Germany; ^2^Center for Individual Development and Adaptive Education of Children at Risk (IDeA)Frankfurt am Main, Germany; ^3^Department of Educational Psychology, Institute for Psychology, Goethe-UniversityFrankfurt am Main, Germany

**Keywords:** approximate number system, non-symbolic numerical comparison, arithmetic, development, elementary school

## Abstract

The approximate number system (ANS) is assumingly related to mathematical learning but evidence supporting this assumption is mixed. The inconsistent findings might be attributed to the fact that different measures have been used to assess the ANS and mathematical skills. Moreover, associations between the performance on a measure of the ANS and mathematical skills may be discontinuous, i.e., stronger for children with lower math scores than for children with higher math scores, and may change with age. The aim of the present study was to examine the development of the ANS and arithmetic skills in elementary school children and to investigate how the relationship between the ANS and arithmetic skills develops. Individual markers of children's ANS (internal Weber fractions and mean reaction times in a non-symbolic numerical comparison task) and addition skills were assessed in their first year of school and 1 year later. Children showed improvements in addition performance and in the internal Weber fractions, whereas mean reaction times in the non-symbolic numerical comparison task did not change significantly. While children's addition performance was associated with the internal Weber fractions in the first year, it was associated with mean reaction times in the non-symbolic numerical comparison task in the second year. These associations were not found to be discontinuous and could not be explained by individual differences in reasoning, processing speed, or inhibitory control. The present study extends previous findings by demonstrating that addition performance is associated with different markers of the ANS in the course of development.

## Introduction

Approximate number representations enable us to discriminate between sets of different numerical quantities, a crucial ability for everyday life. Similar to our performance in discriminating physical dimensions like line length or pitch (e.g., Henmon, 1906), comparing numerical magnitudes is ratio-dependent. We are faster and more accurate in comparing dot arrays with respect to their quantity the smaller the ratio between them is (when dividing the smaller numerosity by the larger one; e.g., van Oeffelen and Vos, 1982). The ability to discriminate between different numerical quantities is present early in life and undergoes a progressive refinement throughout development: in their first hours of life, infants seem to be sensitive to a ratio of 1:3 (Izard et al., 2009) and the precision increases to a ratio of about 9:10 or 10:11 at the age of 20 years (Halberda and Feigenson, 2008). Besides, animals such as monkeys or fish also seem to be able to represent and compare numerical quantities showing similar performance patterns as human adults (Cantlon and Brannon, 2006; Agrillo et al., 2012). This suggests the existence of an evolutionary ancient, innate system, the approximate number system [ANS; see Piazza (2010), for an overview].

It is assumed that the ANS encodes numerosities as analog magnitudes that can be modeled as overlapping Gaussian distributions of activations on a logarithmically compressed internal continuum (Dehaene et al., 2003; Piazza et al., 2004; see e.g., Gallistel and Gelman, 1992, for a different view). Due to the logarithmic compression, overlap between numerosities increases with magnitude, which concurs with a decrease in discriminability. An established measure of the ability to discriminate numerosities and therefore of the precision of the internal representation is the so-called “internal Weber fraction,” which reflects the width of the Gaussian distributions. The Weber fraction measures the smallest numerical difference that can be reliably detected, and equals the difference between the two numerosities divided by the smaller numerosity [e.g., 1:3, (3 − 1)/1 = 2; 7:8, (8 − 7)/7 = 0.14].

ANS precision does not only vary across development but also between individuals of the same age and it has been hypothesized that these inter-individual differences are linked to mathematical skills (e.g., Halberda et al., 2008). However, evidence supporting this proposal is inconsistent. While a number of studies showed that inter-individual differences in performance on a measure of the ANS are related to concurrent and future mathematics achievement, other studies failed to find such relationships (see De Smedt et al., 2013, for an overview). These divergences might be attributed to the fact that different measures have been used to assess the ANS. The range of applied measures includes the internal Weber fractions, mean error rates (ER), mean or median reactions times (RT) as well as distance or ratio effects calculated on the basis of ER or RT in magnitude comparison tasks. According to De Smedt et al. (2013), however, it is not easy to distinguish studies that *have* from those studies that *have not* found significant relationships between the performance on a measure of the ANS and mathematical skills on the basis of the ANS measure employed. In the case of examining children, those studies that used the internal Weber fractions as dependent measure predominantly detected associations with mathematical skills (see Table 1 in De Smedt et al., 2013). Recent studies, however, suggest that these associations are limited to trials of non-symbolic numerical comparison tasks in which the size of the area occupied by the stimuli conflicts with the number of elements (i.e., more numerous stimuli occupy a smaller area). Hence, it was inferred that the association represents an artifact of the inhibitory control demands of theses trials and it could be demonstrated that the correlation became non-significant when controlling for inhibitory control (Gilmore et al., 2013; Wagner Fuhs and McNeil, 2013). Besides different measures assessing the ANS, another possible explanation for the inconsistent findings may be the measure used to assess mathematical skills. Typically, standardized or curriculum measures of mathematics achievement have been employed assessing a range of different mathematical competences. De Smedt et al. (2013) argue that the ANS might, however, be more important for some aspects of mathematical competencies than others, and therefore, associations with specific measures of mathematical performance need to be explored. As indicated by a recent study, associations may also be discontinuous, i.e., stronger for children with lower math scores than for children with higher math scores (see Bonny and Lourenco, 2013). Furthermore, the associations may change with age. As, however, most of the studies looking for associations between the performance on a measure of the ANS and mathematical skills are cross-sectional, potential intra-individual changes have not been revealed. To our knowledge, there is only one longitudinal study examining the development of the association between the ANS and mathematical skills (see Libertus et al., 2013). Individual markers of preschool children's ANS (i.e., the internal Weber fractions and mean RT in a non-symbolic numerical comparison task) and mathematical skills (i.e., counting, comparison of spoken number words, reading Arabic numerals, as well as mental and written calculation) were assessed twice, with a 6-month delay, and improvements in all measures could be detected. Moreover, associations between the ANS and mathematical skills were found at both time points and the ANS was found to predict math ability even when controlling for individual differences in math ability at the initial testing.

**Table 1:** **Comparison of first and second-year performance (paired-sample *t*-tests) with respect to the internal Weber fractions (w comparison), reaction times (in ms) in the non-symbolic numerical comparison task (RT comparison), the addition task, reaction times (in ms) in the visual detection task (proc. speed) as well as reaction times (in ms), omission and commission errors (in %) in the inhibitory control task (RT inhib., ER om. inhib., ER com. inhib.)**.

	**First year**			**Second year**			***p* (Two-sided)**
	***M***	***SD***	***SE***	***M***	***SD***	***SE***	
w comparison	0.35	0.14	0.02	0.25	0.10	0.01	*p* < 0.001
RT comparison	1362	328	40	1321	261	32	*p* = 0.25
Addition	51	22	2.7	70	21	2.5	*p* < 0.001
proc. speed^a^	510	96	12	507	98	12	*p* = 0.79
RT inhib.^a^	730	57	7	697	62	8	*p* < 0.001
ER om. inhib.^a^	7.9	7.0	0.86	5.9	5.6	0.68	*p* < 0.05
ER com. inhib.^a^	5.2	5.2	0.64	5.0	4.4	0.54	*p* = 0.75

n = 67;

a n = 66.

The aim of the present study was to expand this evidence by investigating elementary school children. Indeed, evidence on the intra-individual development of the ANS in elementary school children is missing and it is still unclear how the relationship between the ANS and mathematical skills develops in these children. In order to address these issues, we assessed individual markers of children's ANS and mathematical skills in their first year of school and 1 year later. We used the internal Weber fractions and mean RT in a non-symbolic numerical comparison task as markers of the ANS and decided to concentrate specifically on children's performance in addition tasks as addition represents an essential mathematical skill children learn in the first years of elementary school. To assure that possible associations could not be explained by individual differences in more general performance factors, reasoning abilities were also assessed. Moreover, a visual detection task was used to rule out that possible associations could solely be ascribed to individual differences in general processing speed, what might be the case for an association between mean RT in the non-symbolic numerical comparison task and addition skills. As recent studies suggest that a relationship between the performance on a measure of the ANS and mathematical skills might be an artifact of inhibitory control demands (Gilmore et al., 2013; Wagner Fuhs and McNeil, 2013), a visual Go/NoGo task was used to assess inhibitory control.

## Materials and methods

### Participants

Sixty-seven children (35 girls and 32 boys) completed all tasks at both measurement time points. At the first measurement time point, all children were first-graders (mean age: 87 months; range: 79–94 months) and at the second measurement time point second-graders (mean age: 98 months; range: 90–106 months). The average delay between individual measurement time points was 355 days. Written and informed consent was obtained from all parents involved.

### Materials

All tasks were carried out individually. Apart from the measure of reasoning abilities which was administered only at the second measurement time point, all tasks were carried out at both measurement time points.

### Non-symbolic numerical comparison

Sets of black dots were presented in two white circles on the left and the right hand side of the screen of a 14-inch notebook running Presentation® software (Neurobehavioral Systems, Inc.). From a viewing distance of about 60 cm, each of the white circles had a visual angle of 7.82^°^ (82 mm) and the black dots ranged between 0.10 and 0.14^°^ (1–1.5 mm). On each trial, one of the white circles contained 32 dots (reference numerosity) and the other one 20, 23, 26, 29, 35, 38, 41, or 44 dots (deviants). Each of these eight comparison pairs appeared eight times, four times with the reference numerosity on the left and four times on the right hand side. Every single comparison pair had a unique configuration of dots. In half of the 8 trials per comparison pair, the size of the area occupied by the dots in each circle was held constant (luminance-controlled trials), while in the other half, individual dot size in each circle was held constant (size-controlled trials). Children were asked to indicate without using counting strategies, the side of the larger numerical magnitude by answering with the left index finger when it was larger on the left hand side and by using the right index finger when it was larger on the right hand side. Responses were given by pressing the left and right CTRL-buttons of the notebook's keyboard. RT and ER were recorded, and the instruction stressed both speed and accuracy. The order of trials was pseudo-randomized so that there were no consecutive identical comparison pairs. The experiment started with eight warm-up trials to familiarize children with the task (data not recorded), followed by a total of 64 experimental trials (8 comparison pairs × 2 perceptual control conditions × 4 repetitions). A trial started with the presentation of a black screen for 700 ms. After the black screen had vanished, the target appeared until a response was given, but only up to a maximum duration of 4000 ms. No feedback regarding the correctness of responses was provided. Mean RT and internal Weber fractions were used as individual markers of the ANS (see Halberda et al., 2012; Libertus et al., 2013). The internal Weber fractions were calculated based on ER for eight different ratios (20/32, 23/32, 26/32, 29/32, 35/32, 38/32, 41/32, and 44/32) following the methods described in the Supplemental Data from Piazza et al. (2004). The calculation was based on the formula *y* = 0.5^*^(1 + erf (log (x) / (sqrt (2)^*^ w))), where *y* is the probability of responding “larger” and *x* are the different ratios.

### Addition

We used a subtest of the standardized German scholastic achievement test for mathematics (DIRG; Grube et al., 2010) that includes 110 simple addition problems in which two single-digit numbers (excluding 0 and 1) have to be added. Solutions range from 5 to 10 and ties (e.g., 4 + 4) are not included. The 110 addition problems consist of 24 different problems presented in pseudo-randomized order ensuring that neither identical nor commutated problems follow each other directly. The repetition rate of the different tasks varies (some problems are only presented three times, while others are presented up to six times). The problems were presented in written form on four different pages. Children were asked to write down as many solutions as possible in 4 min adhering to the order of the pages. Addition performance was calculated as the number of correctly answered problems. Total scores ranging from 0 to 110 are reported for each child.

### Reasoning

Raven's Colored Progressive Matrices (CPM; Bulheller and Häcker, 2002) were used to assess inductive reasoning. The CPM is an untimed power test consisting of 36 colored diagrammatic puzzles, each with a missing part which has to be identified from a choice of six. Total scores ranging from 0 to 36 are reported for each child.

### Processing speed

A visual detection task was used to assess individual processing speed. Children were instructed to press the space bar of the notebook's keyboard as fast as possible whenever an “×” appeared in the center of the screen. The target appeared until a response was given, but only up to a maximum duration of 3000 ms. The task comprised 30 experimental trials with varying inter-trial intervals (2000, 3500, 5000, 6500, or 8000 ms). Mean RT is reported for each child.

### Inhibitory control

A visual Go/NoGo task was used to assess inhibitory control. Children were instructed to press the space bar of the notebook's keyboard as fast as possible whenever an “×” appeared in the center of the screen (Go-trials) and to inhibit responses whenever an “+” appeared in the center of the screen (NoGo-trials). The target appeared until a response was given, but only up to a maximum duration of 3000 ms. The task comprised 40 experimental trials (20 Go-trials and 20 NoGo-trials) with varying inter-trial intervals (2000, 3500, 5000, 6500, or 8000 ms). The order of trials was pseudo-randomized so that there were no more than three consecutive identical trials. Mean RT, mean commission ER (button presses in NoGo-trials), and mean omission ER (no button presses in Go-trials) are reported for each child.

## Results

Only trials with correct responses were used for computing mean RT in the non-symbolic numerical comparison task, in the visual detection task, and in the inhibitory control task. Trials in which the response was either given too late (after 4000 ms in the non-symbolic numerical comparison task and after 3000 ms in the visual detection task as well as in the inhibitory control task) or not at all were classified as errors. Responses below 200 ms were excluded from further analysis. This resulted in 0.06% of response exclusions in the non-symbolic numerical comparison task, in 0.22% of response exclusions in the visual detection task, and in 0.06% of response exclusions in the inhibitory control task. Mean ER in the visual detection task was low (first year: 1.5%; second year: 2%) and not further analyzed. Pearson correlation coefficients were computed for the observed variables. To assure that possible correlations between individual markers of the ANS and addition performance within the respective years could not be explained by individual differences in more general performance factors or in inhibitory control, partial correlations were computed controlling for reasoning abilities which were only assessed in the second year, as well as for processing speed and inhibitory control (mean RT, omission and commission ER) of the respective year. Correlations examining the predictive value of markers of the ANS for addition performance of the second year were controlled for reasoning abilities as well as for processing speed, inhibitory control, and addition performance of the first year. Moreover, correlations between the internal Weber fractions of the second year and addition performance of the first year were controlled for the internal Weber fractions of the first year as well as for reasoning abilities, processing speed, and inhibitory control of the second year and correlations between mean RT in the non-symbolic numerical comparison task of the second year and addition performance of the first year were controlled for mean RT in the non-symbolic numerical comparison task of first year as well as for reasoning abilities, processing speed, and inhibitory control of the second year. In the partial correlation analyses including performance in the visual detection and the inhibitory control task at the second measurement time point, one child had to be excluded because of failing to complete these tasks. To test for the possibility that correlations between individual markers of the ANS and addition skills may be stronger for children with lower addition scores than for children with higher addition scores (see Bonny and Lourenco, 2013), we conducted segmented regression analyses using the software SegReg (Oosterbaan, 2011). These analyses allowed us to look for possible breakpoints in the addition performance where the relation with the markers of the ANS changes abruptly. We looked for models with two lines with different slopes or models with a sloping segment followed by a horizontal line. Evidence for a breakpoint would be reflected in greater explained variance compared with a single linear model.

Significant improvements were observed for the internal Weber fractions, the addition performance, mean RT, and mean omission ER in the inhibitory control task but not for mean commission ER in the inhibitory control task, mean RT in the visual detection task, and mean RT in the non-symbolic numerical comparison task (see Table 1).

**Figure 1:**
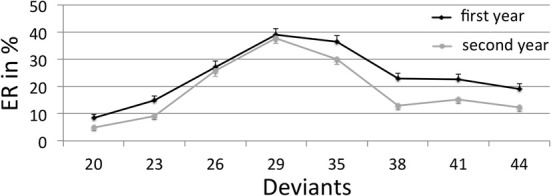
**Mean error rates (ER) and standard errors in % in the non-symbolic numerical comparison task, separately for the different measurement points (first year, second year) as a function of the different deviants (reference numerosity = 32)**.

In the non-symbolic numerical comparison task, ER increased as the ratio between the two to-be-compared numerosities increased: significant linear trends for deviants smaller than the reference [20 vs. 23 vs. 26 vs. 29; first year: *F*_(1, 66)_ = 183.14; *p* < 0.001; second year: *F*_(1, 66)_ = 230.97; *p* < 0.001] and for deviants larger than the reference [35 vs. 38 vs. 41 vs. 44; first year: *F*_(1, 66)_ = 38.96; *p* < 0.001; second year: *F*_(1, 66)_ = 36.61; *p* < 0.001] were found in both years (see Figure 1). In order to look for differences between luminance-controlled and size-controlled trials, we computed the internal Weber fractions and mean RT for both conditions separately. Because of a very low fitting parameter (*R*^2^ < 0.2) of the procedure to calculate the internal Weber fractions, nine children had to be excluded in the first year and 11 children in the second year. As a consequence, we used mean ER as a proxy for the internal Weber fractions (see Mazzocco et al., 2011, for a similar approach; mean ER and the internal Weber fractions for all trials were highly correlated in both years: first year: *r* = 0.95; *p* < 0.001 [two-sided]; second year: *r* = 0.94; *p* < 0.001 [two-sided]) which allowed us to compare the performance in luminance-controlled and size-controlled trials in all participants. Considering mean ER, a significant difference between luminance-controlled and size-controlled trials was found in the second year (mean ER luminance-controlled = 21% vs. mean ER size-controlled = 16%, *p* < 0.001 [two-sided]) and a trend toward a significant difference in the first year (mean ER luminance-controlled = 25% vs. mean ER size-controlled = 23%, *p* = 0.11 [two-sided]). However, in the first and in the second year, ER increased as the ratio between the two to-be-compared numerosities increased for both luminance-controlled and size-controlled trials (significant linear trends for deviants smaller than the reference [20 vs. 23 vs. 26 vs. 29; luminance-controlled—first year: *F*_(1, 66)_ = 97.10; *p* < 0.001; second year: *F*_(1, 66)_ = 117.39; *p* < 0.001; size-controlled—first year: *F*_(1, 66)_ = 98.72; *p* < 0.001; second year: *F*_(1, 66)_ = 230.97; *p* < 0.001] and for deviants larger than the reference [35 vs. 38 vs. 41 vs. 44; luminance-controlled—first year: *F*_(1, 66)_ = 21.10; *p* < 0.001; second year: *F*_(1, 66)_ = 11.76; *p* = 0.001; size-controlled—first year: *F*_(1, 66)_ = 22.62; *p* < 0.001; second year: *F*_(1, 66)_ = 37.93; *p* < 0.001]). Mean RT in luminance-controlled and size-controlled trials did not significantly differ in both years (first year: mean RT luminance-controlled = 1337 ms vs. mean RT size-controlled = 1324 ms, *p* = 0.43 [two-sided]; second year: mean RT luminance-controlled = 1320 ms vs. mean RT size-controlled = 1304, *p* = 0.40 [two-sided]).

**Figure 2:**
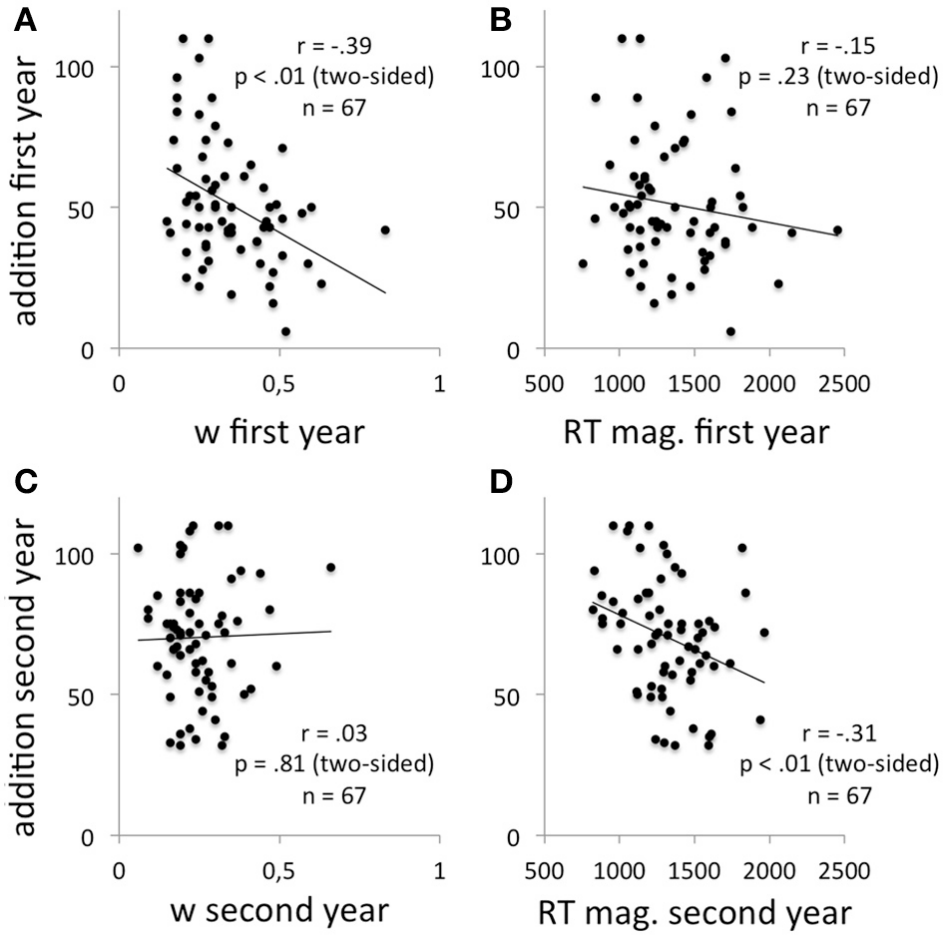
**Correlations between the internal Weber fractions and the addition performance in the first year (A) and in the second year (C) as well as correlations between mean RT in the non-symbolic numerical comparison task and the addition performance in the first year (B) and in the second year (D)**.

Correlation coefficients for the observed variables are shown in Table 2. Significant positive correlations were found between the variables that had been assessed twice, with the exception of the internal Weber fractions. Significant correlations in the bivariate and in the partial correlation analyses were found between the internal Weber fractions and the addition performance in the first year (similarly, mean ER in the non-symbolic numerical comparison task and addition performance correlated significantly—bivariate: *r* = −0.38; *p* = 0.001 [two-sided]; partial: *r* = −0.36; *p* < 0.01 [two-sided]) as well as between mean RT in the non-symbolic numerical comparison task and the addition performance in the second year (see Figure 2). There was no trade-off between mean RT and mean ER in the non-symbolic numerical comparison task (first year: *r* = −0.11; *p* = 0.37 [two-sided]; second year: *r* = −0.01; *p* = 0.94 [two-sided]). Significant correlations in the bivariate and in the partial correlation analyses across the 2 years were only found between addition performance in the first year and mean RT in the non-symbolic numerical comparison task in the second year. A significant correlation between the internal Weber fractions of the first year and addition performance of the second year was only found in the bivariate but not in the partial correlation analyses.

**Table 2:** **Bivariate (below the diagonal) correlation coefficients for all observed variables and partial (above the diagonal) correlation coefficients between individual markers of the ANS (internal Weber fractions [w comparison] and mean reaction times [RT comparison] in the non-symbolic numerical comparison task) and addition performance within and across the respective years (processing speed [proc. speed]; reaction times, omission, and commission errors in the inhibitory control task [RT inhib., ER om. inhib., ER com. inhib.])**.

	**1**	**2**	**3**	**4**	**5**	**6**	**7**	**8**	**9**	**10**	**11**	**12**	**13**	**14**	**15**
1. Addition first year	–		−0.39^*^	0.18^a^	−0.01	−0.33^a^^**^									
2. Addition second year	0.75^^**^^	–	−0.02	0.08^a^	−0.10	−0.30^a^^*^									
3. w comparison first year	−0.39^*^	−0.27^*^	–												
4. w comparison second year	0.03	0.03	0.15	–											
5. RT comparison first year	−0.15	−0.17	−0.10	0.16	–										
6. RT comparison second year	−0.38^**^	−0.31^*^	0.20	0.03	0.54^**^	–									
7. proc. Speed first year	−0.32^**^	−0.25^*^	0.15	0.08	0.26^*^	0.27^*^	–								
8. proc. speed second year	−0.28^*^	−0.18	0.19	0.27^*^	0.08	0.29^*^	0.26^*^	–							
9. RT inhib. first year	−0.30^*^	−0.20	0.15	0.12	0.31^*^	0.35^**^	0.42^**^	0.23	–						
10. RT inhib. second year	−0.26^*^	−0.19	0.23	0.13	0.30^*^	0.47^**^	0.17	0.56^**^	0.36^**^	–					
11. ER com. inhib. first year	0.03	0.01	0.07	0.13	−0.33^*^	−0.30^*^	−0.15	0.07	−0.31^*^	−0.16	–				
12. ER com. inhib. second year	0.01	0.07	0.01	0.31^*^	−0.16	−0.18	0.19	−0.07	0.01	−0.36^**^	0.26^*^	–			
13. ER om. inhib. first year	−0.31^*^	−0.21	0.28^*^	0.20	0.38^**^	0.35^**^	0.61^**^	0.23	0.49^**^	0.30^*^	−0.04	0.04	–		
14. ER om. inhib. second year	−0.19	−0.07	0.33^**^	0.31^*^	0.18	0.27^*^	0.31^*^	0.51^**^	0.29^*^	0.28^*^	0.06	0.11	0.48^*^	–	
15. Reasoning second year	0.40^**^	−0.44^**^	−0.01	−0.01	−0.12	−0.09	−0.22	−0.31^*^	−0.26^*^	−0.20	−0.14	0.03	−0.25^*^	−0.18	–

n = 67;

a n = 66;

* p < 0.05 (two-sided);

** p < 0.01 (two-sided).

Regarding the correlation between the internal Weber fractions and the addition performance in the first year, a model with a breakpoint in the addition performance and two lines with different slopes did not fit the data but a model with a breakpoint in the addition performance and a sloping segment followed by a horizontal line did [*F*_(3, 63)_ = 3.03, *p* < 0.05]. This model did, however, explain less variance than a model without a breakpoint in the addition performance [*F*_(1, 65)_ = 11.99, *p* = 0.001]. For the correlation between mean RT in the non-symbolic numerical comparison task and the addition performance in the second year, neither a model with two lines with different slopes nor a model with a sloping segment followed by a horizontal line fit the data.

As a significant difference between ER in luminance-controlled and size-controlled trials in the second year and a trend toward a significant difference in the first year was observed, we computed correlations with the addition performance separately for both conditions. Significant correlations with the addition performance in the first year could be found for size-controlled ER (bivariate: *r* = −0.41; *p* = 0.001 [two-sided]; partial: *r* = −0.39; *p* < 0.01 [two-sided]) and trends toward significant associations for luminance-controlled ER (bivariate: *r* = −0.21; *p* = 0.09 [two-sided]; partial: *r* = −0.20; *p* = 0.13 [two-sided]). Comparing the correlation coefficients for the luminance-controlled and the size-controlled ER (Hotelling–Williams test; see Steiger, 1980) did not reveal any significant differences (bivariate: *r* = −0.41 vs. *r* = −0.21; *p* = 0.14 [two-sided]; partial: *r* = −0.39 vs. *r* = −0.20; *p* = 0.16 [two-sided]). In the second year, no significant correlations were found (bivariate—ER luminance-controlled: *r* = −0.05; *p* = 0.66 [two-sided], ER size-controlled: *r* = 0.02; *p* = 0.90 [two-sided]; partial—ER luminance-controlled: *r* = 0.20; *p* = 0.12 [two-sided], ER size-controlled: *r* = 0.03; *p* = 0.82 [two-sided]). Comparing the correlation coefficients for the luminance-controlled and the size-controlled ER again revealed no significant differences (bivariate: *r* = −0.05 vs. *r* = 0.02; *p* = 0.65 [two-sided]; partial: *r* = 0.20 vs. *r* = 0.03; *p* = 0.28 [two-sided]).

## Discussion

In the present study, the development of the ANS and addition skills was examined in children in their first 2 years of elementary school. Significant improvements in addition performance and in the internal Weber fractions were found, while mean RT in the non-symbolic numerical comparison task remained unchanged. The developmental change in the internal Weber fractions (from 0.35 to 0.25) is in line with previous findings from cross-sectional studies (see Piazza, 2010). The internal Weber fractions were associated with children's addition performance at the first measurement time point. This association was not found to be non-linear (e.g., stronger for children with lower addition scores than for children with higher addition scores) and it could not be explained by individual differences in reasoning, processing speed, and inhibitory control. At the second measurement time point, however, no association was found between the same measures. Likewise, the internal Weber fractions of the first year were not correlated with the internal Weber fractions of the second year. This might be due to the fact that mean ER in the non-symbolic numerical comparison task, used as a proxy for the internal Weber fractions, was significantly higher in luminance-controlled trials than in size-controlled trials in the second year, whereas no significant difference was found in the first year. The difference between luminance-controlled and size-controlled trials detected in the second year concurs with previous findings (e.g., Wagner Fuhs and McNeil, 2013) and might be related to the fact that luminance and the number of elements are positively correlated in size-controlled trials and uncorrelated in luminance-controlled trials. The visual characteristics of the stimuli could thus, provide an additional cue to number in size-controlled trials, whereas the visual characteristics of the stimuli in luminance-controlled trials might even be obstructive because controlling for luminance involves that the more numerous arrays have smaller dots. As a significant difference between ER in luminance-controlled and size-controlled trials was found only in the second but not in the first year, the influence of the visual cues might have differed at the two measurement time points, possibly resulting in the non-significant correlation between the internal Weber fractions of the first and of the second year.

In contrast to the present study, some other studies investigating the association between the performance on a measure of the ANS and mathematical skills incorporated a condition in the non-symbolic numerical comparison task in which luminance and the number of elements was negatively correlated (so-called inverse or incongruent trials), either in addition to a luminance- and a size-control condition (Wagner Fuhs and McNeil, 2013) or instead of a luminance-control condition (see experiment 1 in Gilmore et al., 2013). In both cases, ER differed significantly between the respective conditions and a relationship between performance on a measure of the ANS and mathematical skills was limited to the inverse trials. Hence, it was inferred that this relationship represented an artifact of the inhibitory control demands of the inverse trials and it could be demonstrated that the correlation became non-significant when controlling for inhibitory control. These findings do not correspond to the results of the present study. Indeed, we found that mean ER in the non-symbolic numerical comparison task did not differ significantly between luminance-controlled and size-controlled trials in the first year, and the association between the internal Weber fractions (or mean ER respectively) and children's addition performance detected in the same year was not limited to luminance-controlled trials and did not disappear when controlling for inhibitory control. Previous studies used other tasks to measure inhibitory control (see Gilmore et al., 2013; Wagner Fuhs and McNeil, 2013) and thus, it is possible that associations would have disappeared when using another task. Moreover, an inclusion of inverse trials requiring high levels of inhibitory control in the present study might possibly have provoked significant differences between the respective conditions of the non-symbolic numerical comparison task at the first measurement time point. The absence of such a condition as well as the choice of the inhibitory control task can, however, hardly explain why the association between the internal Weber fractions (or mean ER respectively) and children's addition performance at the first measurement time point was not limited to luminance-controlled trials. We assume that this might be due to different measures of mathematical skills used in the respective studies. Instead of selectively assessing a particular proficiency like addition, Wagner Fuhs and McNeil (2013) as well as Gilmore et al. (2013) used test batteries assessing a range of different skills. The internal Weber fractions (or mean ER respectively) might be specifically related to addition skills in first graders and this relationship does not seem to be an artifact of inhibitory control demands.

While the internal Weber fractions (or mean ER respectively) were found to be related to addition skills at the first measurement time point, children's mean RT in the non-symbolic numerical comparison task was associated with their addition performance at the second measurement time point. This association was not stronger for children with lower addition scores than for children with higher addition scores and it could not be explained by individual differences in reasoning, processing speed, and inhibitory control. Moreover, children's mean RT of the first year were significantly correlated with the mean RT of the second year and mean RT in luminance-controlled trials did not differ from mean RT in size-controlled trials in both years. Consequently, children's performance in solving simple addition tasks seems to be associated with different markers of the ANS in the course of development. This finding contradicts the results of a previous study on preschool children showing that the internal Weber fractions and mean RT in a non-symbolic numerical comparison task were linked to math skills in both of two successive testing sessions (Libertus et al., 2013). According to Halberda et al. (2012), the internal Weber fraction represents an estimate of the ANS's precision while mean RT in a non-symbolic numerical comparison task represents the amount of time it takes individuals to make their decision. Thus, the present findings might indicate that children's addition performance in the first year of school was related to the individual precision of the ANS while addition performance in the second year was related to the individual speed of retrieving approximate number representations. Following the line of argument that the ANS provides semantic representations of numbers (e.g., Dehaene et al., 2003), children might have relied on the ANS during arithmetic problem solving in order to grasp how the magnitudes of the different task solutions (and of the addends) fall in relation to other magnitudes, and whether the solution is appropriate to the task. While not all the children might have grasped this concept with sufficient clarity to adequately process the different addition tasks in the first year, the majority of the children in the second year might have reached the appropriate level of understanding, attributing stronger impact to the speed of retrieval rather than the precision of the representations in the process of solving the addition tasks in the second year. According to this reasoning, the divergent findings by Libertus et al. (2013) might again be attributed to differences in the measures used to assess children's mathematical skills. Libertus et al. (2013) used a test battery involving counting, comparison of spoken number words, reading Arabic numerals, as well as mental and written calculation. Indeed, using the performance of similar tasks such as the comparison of spoken number words as indicator of mathematical skills and the comparison of non-symbolic numerosities as marker of the ANS may increase the chance of detecting a relationship. This may also explain why Libertus et al. (2013) found that individual markers of the ANS predicted mathematical skills at the second measurement time point in preschool, while no reliable evidence for a prediction of arithmetic skills could be detected in the present study. In fact, as arithmetic skills were found to predict mean RT in the non-symbolic numerical comparison task, results of the present study rather point to the reverse direction of influence. Libertus et al. (2013) also reported a similar relationship between mathematical skills at the first measurement time point and the internal Weber fractions at the second measurement time point. Likewise, a recent study revealed that the acquisition of symbolic numbers and arithmetic enhances the precision of the ANS (Piazza et al., 2013). It can thus be assumed that symbolic and non-symbolic numerical thinking enhance one another over the course of development. Looking at developmental trajectories of associations between different markers of the ANS and different mathematical skills might help to better understand what exactly causes the link between the ANS and mathematical performance. In this regard, the present study extends previous findings by demonstrating that the performance in solving simple addition tasks is associated with different markers of the ANS in the course of development.

### Conflict of interest statement

The authors declare that the research was conducted in the absence of any commercial or financial relationships that could be construed as a potential conflict of interest.
